# Repeated Retrieval of Generalized Memories can Impair Specific Autobiographical Recall: A Retrieval Induced Forgetting Account

**DOI:** 10.1037/xge0001028

**Published:** 2021-01-14

**Authors:** Noboru Matsumoto, Satoshi Mochizuki, Laura Marsh, Jun Kawaguchi

**Affiliations:** 1 Division of Psychology, Faculty of Arts, Shinshu University; 2 Faculty of Social Policy and Administration, Hosei University; 3 MRC Cognition and Brain Sciences Unit, University of Cambridge; 4 Graduate School of Informatics, Nagoya University; 5 Department of Psychology, Otemon Gakuin University

**Keywords:** retrieval-induced forgetting, overgeneral memory, autobiographical memory specificity, depression, rumination

## Abstract

Overgeneral autobiographical memory (OGM) refers to the tendency toward increased general memory and reduced specific memory recall, observed in various psychiatric disorders. Previous studies have suggested that inhibitory processes involved in resolving competition between competing memories may reduce memory specificity via retrieval-induced forgetting (RIF). However, it remains unclear whether the repeated retrieval of general memories can induce forgetting of specific memories. We adapted the RIF paradigm to address this question across three experiments. Participants first generated specific memories in response to positively and negatively valenced cue words. They then generated and repeatedly retrieved general memories for half of the cue words. Recall for all of the original specific memories was later tested. Experiment 1 showed that the retrieval practice of general memories reduced the recall of associated specific memories, regardless of cue valence. Experiment 2 demonstrated that this forgetting effect was cue independent, occurring even when novel retrieval cues were used on the final test. Experiment 3 suggested that this effect was competition dependent, finding a greater RIF effect following practice of general memories (high competition) than following a cue-color association task (low competition). These results suggest that repeated retrieval of general memories suppressed specific memory representations through RIF. These findings are discussed in relation to hierarchical models of autobiographical memory, mechanisms that maintain overgeneral memory tendencies, and the role of retrieval in shaping autobiographical memory.

Overgeneral autobiographical memory (OGM) refers to the tendency to recall generalized summaries of one’s personal past (e.g., “I always have fun drinking at parties”), rather than specific events that can be located in certain time and place (e.g., “Erika’s birthday party last Friday night”; Sumner, 2012; Williams et al., 2007). Overgeneral memory tendencies have been reported across various psychiatric disorders, including depression (King et al., 2010; Liu et al., 2013; Ono et al., 2016), posttraumatic stress disorder (PTSD; Barry et al., 2018; Moore & Zoellner, 2007; Ono et al., 2016), bipolar disorder (Scott et al., 2000), schizophrenia (Warren & Haslam, 2007), eating disorder (Dalgleish et al., 2003), and borderline personality disorder (Jones et al., 1999).

Specific autobiographical recall underpins various cognitive skills that are important in everyday functioning and mental health, including problem solving, future planning, cognitive reappraisal, and making accurate judgments about the self and the world (Hitchcock et al., 2017; Jing et al., 2016). Accordingly, OGM is associated with cognitive problems such as poor social problem solving (Goddard et al., 1996) and impaired episodic future thinking (Williams et al., 1996). OGM is also associated with more prolonged depression (Sumner et al., 2010), and suicidal ideation and attempts (Williams, 2014). These findings have generated interest in OGM as a potential therapeutic target for novel therapies, with promising initial results (Barry et al., 2019; Raes et al., 2009). Understanding the mechanisms underlying OGM tendencies is likely to be critical to the development and refinement of these memory-based therapies. Despite a great deal of research on OGM, our understanding of these mechanisms remains limited.

Hauer and Wessel (2006) have suggested that inhibitory processes that suppress specific memories may contribute to OGM tendencies. The authors draw parallels between impaired recall of specific memories and the experimentally observed phenomenon of retrieval-induced forgetting (RIF). RIF refers to the now well established finding that repeated retrieval of one exemplar (e.g., apple) from a categorical cue (e.g., fruit) can cause forgetting of other exemplars (e.g., orange) from the same category on a later recall test (Anderson et al., 1994). This effect has been shown to result from inhibitory forces acting on competing exemplars (orange) during attempts to recall the target exemplar (apple; see Murayama et al. (2014) for a meta-analytic review). Similar processes might contribute to the co-occurring impairments in specific memory recall alongside high levels of overgeneralized ruminative thinking in clinical disorders such as depression.

Although RIF was originally discovered using neutral word pairs as the stimuli (Anderson et al., 1994; for reviews, Murayama et al., 2014; Storm et al., 2015), it has since been reported for a variety of memory modalities, including autobiographical memories (Barnier et al., 2004; García-Bajos & Migueles, 2017; Glynn et al., 2018, 2019; Harris et al., 2010; Stone et al., 2013; Hauer & Wessel, 2006). For example, Barnier et al. (2004) had participants generate autobiographical memories in response to negative, neutral, or positive cues. Their retrieval practice phase involved elaborating further on a subset of memories. On later testing, the authors observed RIF of unpracticed memories from the practiced category, relative to baseline. Similarly, Hauer and Wessel (2006) asked participants to describe “where,” “when,” “who,” and “what” details of autobiographical memories belonging to two broad categories. Retrieval practice of a subset of five memories from a given category (recalling the who and what details when cued with where and when information) led to the forgetting of other autobiographical memories belonging to that category, relative to the unpracticed (baseline) category.

These previous studies have all used specific memories as the experimental materials, and have, therefore, examined the aftereffects of competition between specific event memories during retrieval. Few have considered the possibility that such inhibitory control mechanisms might operate across different levels of specificity, such that the retrieval of generalized memory traces might weaken associated specific memories. This proposal may seem counterintuitive, particularly in the context of hierarchical models of autobiographical memory, in which one might anticipate competition occurring sequentially throughout the search between items at the same level (Conway & Pleydell-Pearce, 2000). However, there is evidence that RIF occurs across different levels of memory, and even across memory systems. Retrieval from semantic memory has, for example, been found to induce episodic forgetting (Bäuml, 2002), and episodic RIF at a semantic level (Starns & Hicks, 2004), the critical factor being the presence of competition during retrieval. The neural substrates of general memories are close to those of specific memory (Grilli & Verfaellie, 2014; Renoult et al., 2012, 2016), suggesting that they may be closely associated enough to trigger inhibition.

The role of memory valence in RIF of autobiographical experiences remains unclear. Several studies have reported RIF of negative autobiographical memories (Barnier et al., 2004; García-Bajos & Migueles, 2017; Harris et al., 2010; Stone et al., 2013), but findings with regard to positive memories have been less consistent. Some report normal RIF of positive memories (Barnier et al., 2004; Stone et al., 2013), while other studies suggest that positive memories might be resistant to RIF (García-Bajos & Migueles, 2017; Glynn et al., 2018; Harris et al., 2010; Hauer & Wessel, 2006). Evidence even suggests that RIF of negative memories may contribute to the positivity biases that characterize healthy autobiographical memory and are absent in depression (Storm & Jobe, 2012; Marsh et al., 2019), though it remains unclear whether this is a result of nonuniform inhibition, or an amplification of a preexisting bias toward positive retrieval. Understanding the role of memory valence is particularly crucial when considering the clinical relevance of RIF, and its potential relationship with OGM. For example, can rumination on overgeneral negative content (Watkins & Roberts, 2020) induce the forgetting of specific positive memories that might normally serve to place boundaries on negative self-generalizations (Hitchcock et al., 2017)? The present study aims to address this issue by comparing RIF of positively and negatively cued autobiographical memories across varying levels of specificity.

## Aims of the Present Study

**Figure 1 fig1:**
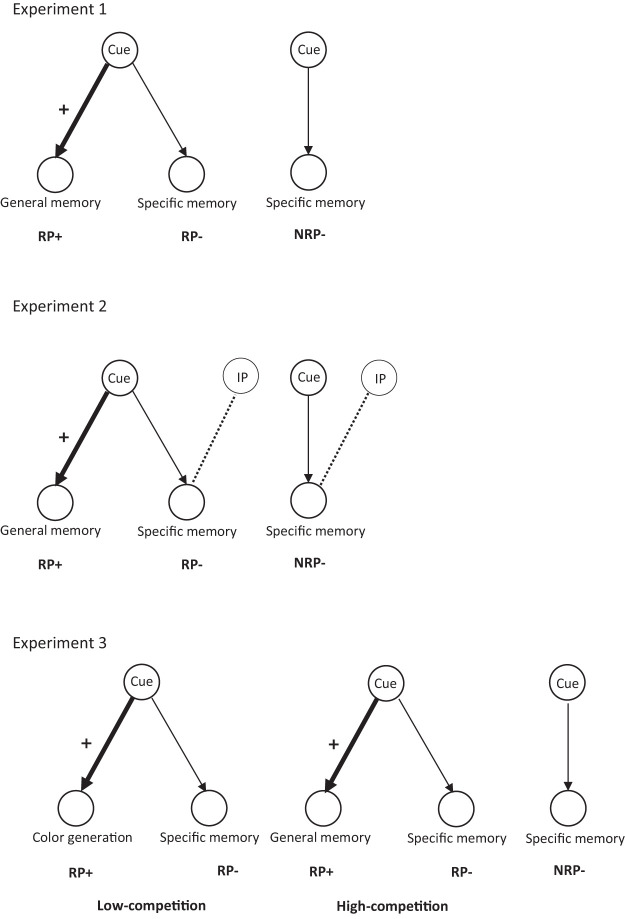
Illustration of the Relationship Constructed Between Cues and Autobiographical Memories for Experiments 1, 2, and 3 *Note*. RP+ refers to memories that were practiced (and/or generated) during the retrieval practice phase. RP− refers to unpracticed memories that were associated with practiced cues. NRP− refers to items that were unpracticed memories attached to unpracticed cues, which served as a baseline condition. In Experiment 2, an independent probe (IP) was used to assess final recall of RP− and NRP− items. In Experiment 3, general memory retrieval served as a high-competitive condition and color generation served as a low-competitive condition.

Across three experiments, we aimed to examine whether RIF arises not only from the competition between specific autobiographical memories, as previously shown (e.g., Barnier et al., 2004), but also from competition between general and specific memories. In Experiment 1, we examined whether the generation and repeated retrieval of general memories can lead to forgetting of associated specific autobiographical memories. Experiment 2 investigated whether the forgetting of specific memories was a result of inhibition during retrieval, or whether it could be explained by interference mechanisms (Murayama et al., 2014). Although these studies were conducted in healthy student samples, we checked for any influence of depression symptoms and rumination levels, because there is some evidence to suggest that psychiatric symptoms impact RIF (Groome & Sterkaj, 2010; Whitmer & Banich, 2010). Experiment 3 was performed to exclude the possibility that the RIF effect arises from the generation of any new cue-associate (generating a color associated with the retrieval cue), and to verify that the forgetting is dependent on competition between related general memory and specific memories. Finally, due to the aforementioned inconsistent findings with regards to the role of valence in RIF, we examined the effect of cue-valence on forgetting. An overview of the procedures in all of the studies is provided in Figure 1.[Fig fig1]

## Experiment 1

### Method

#### Participants

Thirty Japanese undergraduate and graduate students (16 female, *M* = 22.20 ± 2.30 years old) were recruited from the University of Tsukuba and Nagoya University. This study was approved by the ethical committee of Human Sciences, University of Tsukuba (Approval No. 28–36), and by the Graduate School of Informatics, Nagoya University (Approval No. 170831–2-01).

#### Questionnaires

The Beck Depression Inventory-Second Edition (BDI-II; Beck et al., 1996; Japanese version Kojima & Furukawa, 2003) was administered to measure depressive symptoms, and the Ruminative Responses Scale (RRS; Treynor et al., 2003; Japanese version: Hasegawa, 2013) was used to measure rumination levels. Both BDI-II (Cronbach’s alpha = .91) and RRS (Cronbach’s alpha = .83) showed high internal consistency. BDI scores range from 0–63, and RRS scores range from 22–88, with higher scores reflecting higher symptom levels.

#### Autobiographical Memory RIF Paradigm

##### Elicitation Phase

The participants were given the definition of a specific memory (i.e., an event that happened at a particular time and place). They were then presented with 30 positive and 30 negative cue words and asked to select 20 (10 positive, 10 negative) that reminded them of a specific memory. Participants were not explicitly instructed that these lists contained positive or negative words. The full pool of 60 cue words are listed in the Appendix. Both the memory and the cue word were recorded in a Microsoft Excel file by the experimenter for use in the later learning phase on a Psychopy program (Version 1.90.03; Peirce et al., 2019). If the content of a specific memory contained its cue word, the experimenter made edits that omitted the cue word. The only constraints were that the memory had to be specific, and participants were not allowed to use the same word twice. Based on Raes et al. (2007), reported memories were classified as specific memory for events that occurred at a specific time and place and lasted less than a day, general memory for memories that summarized similar events or lasted more than a day, and semantic association for semantic memories that were not events. If a nonspecific memory was reported, as determined by a trained experimenter, the participant was prompted to recall a specific memory. Participants also rated the emotional valence of each memory using a 5-point Likert scale (1 = *negative*, 5 = *positive*) and vividness using a 7-point scale (1 = *cloudy and imageless*, 7 = *clear and vivid*) for each memory recall.

##### Learning Phase

The participants were asked to learn the associations between the 20 cue words and the specific memories generated in the elicitation phase. Each cue word was displayed once for 7 s on the computer screen alongside the written description of the associated memory that was recorded by the experimenter in the elicitation phase.

##### Retrieval Practice Phase

The retrieval practice phase involved two stages: (a) the generation of general memories for a subset of cue words, and (b) repeated retrieval of those memories when prompted with the associated cue. Half of the cue words from the learning phase (5 positive and 5 negative cues) were used for retrieval practice. Within each valence condition, the cue words were randomly assigned to the retrieval practice condition and baseline condition. First, the participants were given the definition of a general memory (i.e., memories that summarize similar events) and an example (“I often walk to my big neighborhood park” in response to the cue “big”). Each of the retrieval practice cue words was then presented on screen one by one, and participants were asked to generate a general memory in response to each of them. If the experimenter judged that a nongeneral memory was reported (according to the classification criteria described in the elicitation phase), participants were asked again to generate a general memory. There was no time limit for this generation phase. Subsequently, each cue word was presented again, and participants were given 30 s to recall the associated general memory. This process was repeated four times. The cue presentation order was pseudorandomized, with alternate presentation of positive and negative cues. There was no feedback during this retrieval practice phase because the strength of the association between Rp+ (retrieval practiced) items and cues has little effect on the RIF (Anderson et al., 2000), and successful recall is not necessary to induce RIF (Storm et al., 2006).

##### Final Recall Test Phase

In the final recall test, the participants had to recall each of the specific memories previously associated with the cue words. The cue words were presented in a pseudorandom order, and participants were given 10 s to begin reporting the memory. This time limitation was imposed to avoid a ceiling effect. If the participants could not report the associated specific memory, the next trial was begun. We regarded the reported memory as a correct recollection if a major part of the narrative corresponded to the learned specific memory. For example, a learned memory of “a customer was lashing out at my co-worker at my part-time job” was considered to be a correct recollection if the participant reported that “a customer yelled at the cashier at her part-time job, saying, ‘You should be fired.’” On the contrary, the learned memory of “I came home last month and ate my mother’s food for the first time in a long time” was considered to be an incorrect recollection if the participant reported that “I was relieved to see that everyone hadn’t changed much when I got home to my parents.” Recall of general memories was not tested, as the study aimed to examine the impact of general memory retrieval practice on later recall of related specific memories.

##### Filler Task

A calculation task was inserted as a 5-min filler immediately after both the learning phase and the retrieval practice phase. The participants were asked to repeatedly subtract 13 from 1,011 in their head after the learning phase and 17 from 2,048 after the retrieval practice phase. Each of the calculation tasks lasted 5 min.

#### Procedure

The experiment was advertised as a study of autobiographical memory. After obtaining informed consent, participants answered the questionnaires and then completed the RIF paradigm. Once all tasks were complete, participants received a debriefing and were paid JPY1,000 (approximately $10) for their participation.

To ensure the accuracy of the experimental manipulation, an independent rater, blinded to the experimental conditions, was asked to categorize the memories from the elicitation and generation phases as semantic, general, or specific. The memories were presented to the independent rater in a randomized order. There was good agreement with the experimental demands (Cohen’s *k* = .81; i.e., where participants were instructed to retrieve specific memories, the independent rater mostly agreed that their response was indeed a specific memory). Another independent rater coded the correctness of memories recalled in the final recall test phase, showing excellent agreement with the experimenter’s judgment (Cohen’s *k* = .97). Since sufficient agreements of memory specificity and final recall were found, subsequent analysis was performed without the exclusion of data.^1^

### Results and Discussion

The mean BDI-II score was 8.20 (*SD* = 7.81), and the mean RRS score was 44.40 (*SD* = 9.38), indicating low levels of depressive symptoms overall.

To confirm that memory valence was successfully manipulated by cue valence, a *t* test was performed. Positive cues elicited memories that were rated by the participant as significantly more positive (*M* = 4.05, *SD* = 0.94) than negative cues (*M* = 2.05, *SD* = 0.85), *t* = 25.05, *p* < .001. Furthermore, we performed a *t* test to confirm the homogeneity of memory assigned to Rp- (unpracticed memories to practiced cues) and Nrp (unpracticed memories to unpracticed cues) conditions and found no difference in vividness, *t* = 0.22, *p* = .82 or emotional intensity, *t* = 0.83, *p* = .41 (which was calculated by the absolute value of the difference from the theoretical mean [3]). Participant-rated memory vividness did not differ with cue valence (*t* = 0.53, *p* = .60; positive: 5.20 ± 1.26; negative: 5.14 ± 1.28). According to the independent rater, participants were able to recall the correct general memories on most retrieval practice trials (89.7% general memories, 0.5% specific memories, 5.4% semantic memories, and 4.3% no memory or an incorrect memory (not the general memory that was originally generated).

**Figure 2 fig2:**
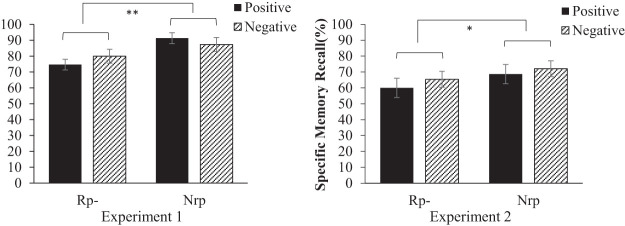
Number of Specific Memories Recalled in Experiments 1 and 2 *Note*. Error bars indicate standard errors. Rp– = cue words used during the retrieval practice; Nrp = cue words not used in the retrieval practice. * *p* < .05. ** *p* < .01.

We compared the proportion of correctly recalled specific memories on the final recall test in response to cue words used during the retrieval practice (Rp− items) with those not used in the retrieval practice (Nrp items). The effect of cue valence was also considered. A two-way analysis of variance (ANOVA) revealed a significant main effect of retrieval practice, *F*(1, 29) = 10.37, *p* = .003, η_*G*_^2^ = .08. As shown in Figure 2, Rp− items were recalled less frequently than the Nrp items (Rp−: 77.35%; Nrp: 89.3%) The main effect of cue valence, *F*(1, 29) = 0.06, *p* = .81, η_*G*_^2^ = .00 and the interaction, *F*(1, 29) = 2.03, *p* = .17, η_*G*_^2^ = .01 were not significant. The size of the RIF effect (Nrp subtracted from Rp−) was not significantly correlated with depressive symptoms, *r* = .16, *p* = .41 or rumination, *r* = .10, *p* = .61 within this healthy sample.[Fig fig2]

In summary, Experiment 1 examined whether repeated retrieval of general memory can induce the forgetting of related specific memories and, if so, whether this depends on the affective valence of cues. Retrieval practice of general memories led to forgetting of specific memories attached to the same cue (Rp− memories), relative to memories for which no associated general memory was practiced (Nrp− or baseline memories). This effect was observed regardless of the affective valence of the cues. To our knowledge, this is the first study showing that RIF can occur across levels of autobiographical memory specificity. These findings are consistent with previous demonstrations of RIF occurring between semantic and episodic memory (Bäuml, 2002; Starns & Hicks, 2004).

Repetitive retrieval of generalized or abstract memories is a central feature of rumination. Williams et al. (2007) suggested that rumination and abstract self-referential thinking contribute to OGM by capturing attention and blocking the retrieval of specific memories. The present findings raise the possibility that this ruminative retrieval style may also have negative consequences in terms of impairing access to related specific memories. However, the extent to which the findings bear relevance to real-world autobiographical memory dynamics, and thus their clinical relevance, hinges, to some extent, on the cue dependence of the effect. In the real world, there are many environmental cues that might activate a memory. If forgetting of specific memories results simply from a disrupted association with the practiced cue, it is likely that over time, these memories will simply be activated by different cues in the environment, and the effects of repetitive retrieval are likely to be minimal. By contrast, if the memory representation itself is weakened in a cue-independent manner, it becomes possible that repetitive retrieval of general memories could directly contribute to deficits in specific memory recall. Experiment 2 addressed this question using the independent probe technique to examine whether the RIF effect generalized to novel retrieval cues, which would indicate suppression of the memory trace itself.

## Experiment 2

While Experiment 1 found that the repetitive retrieval of general memories reduced the recollection of specific memories, it remained unclear whether this RIF effect was an aftereffect of inhibition of those memories during retrieval, or whether it could be explained by interference accounts of RIF (Murayama et al., 2014). Inhibitory accounts of RIF suggest that forgetting results, at least in part, from inhibition of competing Rp− memories during selective retrieval of practiced items. Interference accounts, by contrast, suggest that forgetting occurs due to a strengthening of the relationship between the practiced item and the retrieval cue, which blocks retrieval of unpracticed memories on the final test.

According to interference accounts of RIF, retrieval practice causes no change in the stored representation of the competitor (Rp−) memory, so the targeted memory can be recalled as usual in response to novel cues that were not associated with the practiced item. Conversely, the inhibition account assumes that forgetting of Rp− memories occurs at the level of the memory representation itself, and impaired recall would be expected regardless of the cue used to probe memory on the final recall test. By using novel retrieval cues to probe recall on the final test, one can differentiate between these two possible mechanisms (Anderson & Green, 2001; Wang et al., 2015). Using this independent probe method, Experiment 2 aimed to confirm whether specific memories were forgotten as a result of their inhibition during retrieval practice, or as a result of interference from practiced memories on the final test.

### Method

#### Participants

Thirty Japanese undergraduate and graduate students (19 female, *M* = 19.70 ± 1.95 years old) were recruited from Nagoya University. This study was approved by the ethics committee of the Graduate School of Informatics, Nagoya University (Approval No.170831–2-01).

#### Questionnaires

Before starting the main experiment, participants completed the same set of background questionnaires as in Experiment 1.

#### RIF Paradigm on Autobiographical Memory

##### Elicitation Phase

As in Experiment 1, participants were first given the definition of a specific memory (an event that occurred at a particular time and place). A list of 30 positive words was then presented on the computer screen, and participants were asked to report a specific memory associated with a combination of two cue words. As an example, participants were presented with the memory “When I came to Nagoya University for the first time (I was excited it was so big; in Japanese: 初めて名古屋大学に来たとき,その広大さにワクワクした)” in association with the cues “excited” and “big.” As in Experiment 1, if the memory description contained the cue word, the experimenter edited the description to omit the cue. In the aforementioned example, the phrase in brackets was omitted from the learning phase because it contained the cue words. They continued until they had generated 10 specific memories, as confirmed by the experimenter, not using any cue word twice. Memory descriptions were documented by the experimenter as in Experiment 1 for use in the learning phase. This procedure was repeated for a second list of 30 negative words. The result was a list of 10 specific positive and 10 specific negative memories, each associated with two cue words.

##### Learning Phase

In the learning phase, the participants were asked to learn the associations between each specific memory and one of the two cue words. Each [cue word] – [specific memory description] pair was presented on the computer screen for 7 s. The presented cue word was selected at random and was the cue used for retrieval practice cue (hereafter referred to as the practiced cue). The second cue word, referred to hereafter as the “independent cue,” was not used until the final recall test.

##### Retrieval Practice Phase

As in Experiment 1, retrieval practice was carried out on five positive cue words and five negative cue words from the learning phase. Participants were first asked to generate a general memory in response to each of the presented cue words. They were then asked to recall the same general memories a further four times in response to each cue word appearing on the screen. They were given 30 s to recall the general memory, and if they did not recall the memory within the time limit, the next word was presented.

During the memory generation phase, each memory was reported to the experimenter, who confirmed whether the memory was general before the participant could move on to the next cue. Participants did not receive feedback on their responses during the repeated retrieval phase.

##### Final Recall Test Phase

In the final recall test phase, recall of all 20 specific memories was probed using the independent cues generated in the elicitation phase. Participants were presented with each cue one by one in a random order and were given 10 s to start the reporting of each specific memory. If the participants did not recall the specific memory within the time limit, the next trial was begun.

##### Filler Task

The participants performed a calculation filler task immediately after the learning phase and the retrieval practice phase, as in Experiment 1.

#### Procedure

The participants were told a cover story that they were participating in studies of autobiographical memories associated with multiple emotions. After obtaining informed consent, they completed the questionnaires. Subsequently, the RIF tasks were performed. In all of the tasks, the order of cue presentation was pseudorandomized (i.e., alternate presentation of positive and negative words). Finally, the participants received a debriefing and were given JPY1,000 for their participation. The specificity of memories generated in the elicitation phase and the retrieval practice phase was later assessed by an independent rater, showing good agreement with the experimental demands (Cohen’s *k* = .82). Another independent rater assessed the correctness of memories recalled in the final test, showing high agreement with the experimenter (Cohen’s *k* = .95).

### Results and Discussion

The mean BDI-II score was 10.17 (*SD* = 6.68), and the mean RRS score was 41.80 (*SD* = 12.67), again indicating minimal depression and low levels of rumination.

Experiment 2 did not require participants to rate the emotional valence of their specific memories, but two independent raters categorized each memory as either positive, neutral, or negative. Interrater agreement was high (Cohen’s *k* = .84). Memories with disagreements on the classification were discussed and agreed upon. Overall, 91.3% of specific memories triggered by positive cues were positively valenced and 95.1% of specific memories triggered by negative cues were negatively valenced, suggesting cue valence congruent recall. We next performed a chi-square test to confirm the homogeneity of memory assigned to Rp− and Nrp conditions and found no difference in the proportion of positive, negative, and neutral memories (χ^2^ (2) = 2.87, *p* = .24). As in Experiment 1, we observed that participants were able to retrieve the correct general memories on the majority of trials (93.0%) during the retrieval practice phase (0.8% of specific memories, 3.4% of semantic memories, and 2.9% of no memory or an incorrect general memory: i.e., not the originally generated memory; see Footnote 1). Note that the independent cue word never appeared in the general memory descriptions.

Figure 2 shows the final recall performance across conditions. A 2 (retrieval condition: Rp−/Nrp) × 2 (valence: positive/negative) ANOVA showed a significant main effect of the retrieval condition, *F*(1, 29) = 5.36, *p* = .028, η_*G*_^2^ = .02. Rp− items were recalled at a lower rate than the Nrp items, indicating a significant RIF effect (Rp−: 62.25%; Nrp: 70.35%). The main effect of valence, *F*(1, 29) = 1.79, *p* = .19, η_*G*_^2^ = .01 and the interaction effect, *F*(1, 29) = 0.09, *p* = .76, η_*G*_^2^ = .00 were not significant. Again, the RIF effect was not significantly correlated with depressive symptoms, *r* = .18, *p* = .35 or rumination, *r* = .14, *p* = .46.

These findings extended those of Experiment 1, demonstrating a cue-independent RIF effect. These results suggest that the forgetting of specific memories was, at least in part, due to inhibition during the repeated retrieval of general memories. As such, specific memories appear to have been suppressed at the level of their representation, rather than by disrupted association with the retrieval cue. It should be noted, however, that the effect size of RIF was smaller than in Experiment 1, indicating that interference effects may also have contributed to the forgetting observed in Experiment 1.

## Experiment 3

Experiments 1 and 2 both demonstrate forgetting of specific memories as a result of generating, and then repeatedly retrieving general memories. Experiment 2 demonstrated that this occurs in a cue-independent manner. However, the retrieval practice phases of these experiments involved both (a) the generation of, and (b) the repeated practice of general autobiographical memories. As such, these experiments cannot completely rule out the possibility that simply generating any new cue associate would interfere with the later recall of the specific memory.

Previous studies have found that semantic generation of new items in response to a given retrieval cue can lead to the forgetting of previously learned items associated with the same cue (Bäuml, 2002; Hellerstedt & Johansson, 2014). In these paradigms, the retrieval practice phase involved searching semantic memory for a novel item that fitted a given retrieval cue, suppressing previously studied nontarget items associated with the same cue, leading to their later forgetting. Critically, this effect was found to be competition dependent, indicating an underlying inhibitory mechanism. Specifically, Hellerstedt and Johansson (2014) manipulated competition strength using exemplars of varying taxonomic frequency (high-frequency items were considered stronger). Greater RIF was observed in the high-competition condition, in which participants were cued to generate weak new exemplars, and suppress a strong previously studied exemplar, than in the low-competition condition in which the previously studied exemplar was weak and the newly generated item strong. These findings oppose interference-based accounts of RIF which would predict a strong novel cue-associate to interfere more with the retrieval of a weak previously studied item, instead supporting the inhibitory account, which postulates that suppress a more strongly activated competitor elicits stronger inhibition.

Experiment 3 was designed to address this issue in the context of autobiographical RIF, confirming whether the forgetting of specific autobiographical memories associated with a given retrieval cue was dependent on the generation and retrieval of a competing general memory, or could occur with the generation of any novel cue-associate. The retrieval practice condition used in Experiments 1 and 2 was compared with a color-association condition, in which participants simply had to generate a color in response to the cue word. The latter condition was anticipated to only weakly activate the original specific memory, and, therefore, generate a low level of competition. The memory generation and practice condition, by contrast, requires engagement of the autobiographical memory system for the retrieval search, which is likely to activate the original specific memory associate, triggering greater competition and greater need for inhibition. As such, we predicted greater levels of RIF in the general memory practice condition than the color generation condition.

### Method

#### Participants

The effect sizes of RIF in Experiments 1 and 2 were integrated (*d* = 0.54) to determine the sample size. Twenty-nine participants were required based on the criteria α = .05 and power = 0.80. To satisfy these criteria, we recruited participants from Nagoya University and Shinshu University, resulting in 29 Japanese undergraduate participants (21 female, *M* = 20.28 ± 1.31 years old). This study was approved by the ethics committee of the Graduate School of Informatics, Nagoya University (Approval No. 200812–2-01).

#### RIF Paradigm on Autobiographical Memory

Due to the coronavirus outbreak, this task consisted of a preliminary web-based memory investigation and an online session on Zoom. The initial memory elicitation phase was conducted using Survey Monkey, and the main experiment was conducted with the experimenter running the PsychoPy program and sharing the screen with participants on Zoom.

##### Elicitation Phase

The survey was conducted on Survey Monkey (https://jp.surveymonkey.com/). Participants were asked to describe a specific memory associated with each of 40 cue words (20 positives and 20 negatives) and then to rate the emotional valence of the memories on a 5-point scale (1 = *negative*, 5 = *positive*). The experimenter reviewed their responses and selected 30 memories to be used in the learning phase (the redundancy was included to ensure a sufficient number of specific memories were generated, as the experimenter could not give real-time feedback). Of the memories judged to be specific, those whose emotional valence matched cue valence were prioritized, and the rest were randomly selected. Participants for whom the number of memories judged to be specific was less than the minimum number of memories required for the learning phase were asked to respond again.

##### Learning Phase

Of the cues and specific memories reported in the elicitation phase, 30 pairs (15 positive cues and specific memories and 15 negative cues and specific memories) were used in the learning phase. Each pair was presented in random order for 7 s and participants were asked to learn the associations. Where necessary, the written memory descriptions were edited slightly to ensure that they did not contain the cue word.

##### Retrieval Practice Phase

The retrieval practice phase set three conditions in a within-subject design—general memory condition (high-competition condition), color generation condition (low-competition condition), and baseline condition. 10 cues (5 positives and 5 negatives) were randomly assigned to each condition. Cues assigned to the baseline condition did not appear at all in the retrieval practice phase. Cues assigned to the general memory condition and the color generation condition were presented in random order, and the condition instruction was presented onscreen at the same time. In the general memory condition, participants performed the same task as in Experiments 1 and 2, starting by generating a general memory for each cue and then practicing retrieving those memories when presented with each cue a further four times. In the color generation condition, participants were simply asked to report a color that they associated with the cue word (e.g., red, blue). This was repeated five times. Participants were told that they did not need to refer to their previous responses, and they could report new or old color associations with each round cue presentation. There was no time limit for the first round in either condition, but a 30-s time limit was implemented from the second round onward.

##### Final Recall Test Phase

In the final recall test, recall of all 30 specific memories was probed using the cues learned in the learning phase. To prevent the output interference, we tested cues that were used to recall general memories and to generate colors first and then tested baseline cues that were not used in the retrieval practice phase (i.e., Nrp condition). All other procedures were identical to those in Experiments 1 and 2.

##### Filler Task

The participants performed a calculation filler task immediately after the learning phase and the retrieval practice phase, as in Experiments 1 and 2.

#### Procedure

The experiment was advertised as a study of autobiographical memory. Participants who provided their informed consent were asked to complete the web-based memory elicitation survey the day before the main experiment session. The next day, they completed the main experimental session, including the learning phase, retrieval practice phase, filler task, and final recall test. Participants received a debriefing and were given JPY1,500 for their participation. The specificity of memories used in the study phase and the retrieval practice phase was later assessed by an independent rater, showing that participant responses were largely consistent with the experimental demands (Cohen’s *k* = .94). Another independent rater coded the correctness of memories recalled in the final recall test, showing high agreement with the experimenter (Cohen’s *k* = .95).

### Results and Discussion

As in Experiment 1, cue valence significantly predicted the valence of recalled memories, *t* = 52.85, *p* < .001, suggesting that positive cues triggered positively valenced memory (*M* = 4.48, S*D* = 0.62) and negative cues triggered negatively valenced memory (*M* = 1.85, *SD* = 0.77). Moreover, we performed one-way ANOVA to confirm the homogeneity of memory assigned to general memory, color generation, and baseline conditions and found no differences in emotional intensity, *F* = 1.08, *p* = .34 calculated by the absolute value of the difference from the theoretical mean. Correct general memories were reported for the majority (88.6%) of retrieval practice trials (0.9% of specific memories, 2.8% of semantic memories, and 7.8% of no memory or an incorrect memory [not the general memory that was originally generated]; see Footnote 1).

**Figure 3 fig3:**
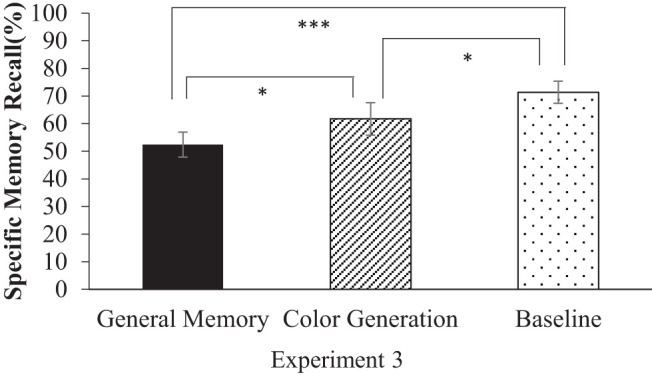
Number of Specific Memories Recalled in Experiment 3 *Note*. Error bars indicate standard errors. * *p* < .05. *** *p* < .001.

Figure 3 shows the proportion of correctly recalled specific memories in the baseline, general memory practice and color generation conditions. A 3 (retrieval condition: general memory/color generation/baseline) × 2 (valence: positive/negative) ANOVA showed a significant main effect of the retrieval condition, *F*(2, 56) = 11.55, *p* = <.001, η_*G*_^2^ = .10. The main effect of valence, *F*(1, 28) = 0.08, *p* = .78, η_*G*_^2^ = .00 and the interaction effect, *F*(2, 28) = 1.90, *p* = .16, η_*G*_^2^ = .02 were not significant. Post hoc tests using the Holm method showed that Participants recalled significantly fewer correct specific memories in the general memory practice condition (52.41%), and in the color generation condition (61.72%) than in the baseline condition (71.38%; *t* = 4.81, *d* = 0.89, *p* < .001 and *t* = 2.45, *d* = 0.45, *p* = .035, respectively). The difference between the general memory practice condition and the color generation condition was also significant (*t* = 2.36, *d* = 0.44, *p* = .035); recall of specific memories was significantly more impaired following general memory practice than following color generation.[Fig fig3]

The differential RIF effect across conditions indicates that the inhibition of specific memories is dependent on the degree to which they compete with retrieval or generation of the target item during the retrieval practice phase. Specific autobiographical memories are likely to be only weakly activated during the color generation task, triggering only low levels of inhibitory control, and a smaller forgetting effect. By contrast, the search and practice of general autobiographical memories are likely to activate competing specific memories more strongly, requiring greater levels of inhibition to overcome the competition. Interference effects are likely to have contributed to the forgetting effect across both the general memory and color association conditions, although the results of Experiment 2 suggest that interference mechanisms are insufficient to fully explain the forgetting observed following general memory practice.

## General Discussion

Across three experiments, the present study examined the impact of repeated retrieval of general memories on related specific memories. In Experiment 1, we showed that repetitive retrieval of general memories reduced recollection of specific memories. In Experiment 2, we demonstrated that this RIF effect was maintained when an independent cue was used on the final recall test, indicating a weakening in specific memory representations themselves, rather than a disrupted association with the cue. This cue independence indicated that forgetting was an aftereffect of inhibitory control recruited during the retrieval search process to resolve competition, although the slightly smaller RIF effect in the independent probe condition indicates that interference also contributed to the forgetting observed on same-probe tests. These RIF effects were not attenuated by higher depressive symptoms or rumination levels within our healthy student sample. Experiment 3 demonstrated that the forgetting effect was dependent on the level of competition during retrieval practice, with significantly greater RIF resulting from the generation and practice of a general autobiographical memory associated with the cue than from the repeated generation of a color association.

While previous studies have shown that RIF occurs between specific autobiographical memories (Barnier et al., 2004) and between episodic and semantic memories (Bäuml, 2002), the present study is the first to demonstrate RIF occurring across levels of specificity within autobiographical memory, with the inhibition of specific autobiographical memories occurring as a result of repeated general memory retrieval.

These findings might seem at odds with hierarchical models of autobiographical memory such as the self-memory system (Conway & Pleydell-Pearce, 2000). One might assume, based on such models, that retrieval of a generalized trace might activate, rather than suppress specific memories that lie beneath it. Indeed, Rp− items that are highly integrated with practiced items are typically resistant to RIF (e.g., Anderson & McCulloch, 1999; Goodmon & Anderson, 2011). However, evidence suggests that autobiographical semantic memory does not prime autobiographical episodic memory recalled from the same cues (Klein et al., 2002). This indicates that specific and general autobiographical memories might not be too highly integrated, and that competition may operate across varying levels of autobiographical memory specificity. The present results support this conclusion; the forgetting of specific memories indicates that competition occurred across levels of specificity, requiring inhibition of the specific memory.

While the present results indicate that retrieval competition and inhibition can occur across hierarchical levels of autobiographical memory specificity, the particular contexts and goals of retrieval are likely to influence the extent to which such memories compete versus coactivate. In the present study, participants were instructed to retrieve general memories during the retrieval practice phase. Should a specific memory have come to mind, this would have constituted an incorrect response and should have been ignored. The goal of retrieving a general memory may, therefore, have been critical to the observed forgetting effect.

The likely goal-dependent nature of this forgetting effect has potential clinical importance. Repetitive rumination and retrieval of abstract, overgeneral memories that characterize many psychiatric disorders are frequently associated with positive metacognitive beliefs about the functions of rumination (Papageorgiou & Wells, 2001; Watkins & Moulds, 2005). Such positive beliefs about rumination may serve to shift retrieval goals in favor of generalized memories, with specific memories perceived as an irrelevant distraction, and thereby suppressed. Relatedly, the avoidance of specific memories, which, during low mood are likely to be negative in nature, is thought to constitute an emotional regulation strategy (Williams et al., 2007). This functional avoidance theory of OGM provides another reason that retrieval goals might shift in favor of general memories, making specific memories a target for inhibition. That such a shift in retrieval goals might reinforce overgeneral retrieval tendencies by weakening specific memories in the longer term has important clinical implications. It is, therefore, important for further research to confirm the circumstances under which general memory retrieval might weaken related specific traces, and establish the extent to which this effect is goal dependent.

The finding that repeated retrieval of overgeneral autobiographical memories can induce forgetting of specific memories raises important concerns about the longer-term impact of rumination on memory accessibility. Overgeneral memory tendencies have recently been reported under conditions of direct (spontaneous) retrieval in depression (Matsumoto et al., 2020) and PTSD (Schönfeld & Ehlers, 2017), as well as under generative conditions involving an active retrieval search. This correlation indicates an imbalance in the availability of specific and general memory traces, rather than simply a deficit in control over the retrieval search process. RIF might offer a candidate mechanism for this shift in memory availability.

Importantly, we observed RIF of both emotionally positive and emotionally negative autobiographical memories. Some previous studies have found RIF only for negative memories, suggesting that positive memories were somehow resistant to RIF (Harris et al., 2010; Hauer & Wessel, 2006). Other studies, however, have found similar levels of RIF regardless of cue valence, consistent with the present study. That positive memories appear to be susceptible to these inhibitory mechanisms may be particularly problematic in the context of low mood. Retrieval of specific positive memories serves to place boundaries on negative self-generalizations, limiting the extent to which they come to define the self (Hitchcock et al., 2017). If rumination on negative content reduces the availability of related specific positive experiences, it may become increasingly difficult to challenge and limit negative generalizations about the self and the world.

Finally, the results of the present study raise the question of whether repetitive retrieval of specific memories can constrain the availability of general memories. Memory specificity training has been developed as an intervention for depression, targeting OGM (Raes et al., 2009; for a meta-analytic review, see Barry et al., 2019). These interventions aim to increase memory specificity by training individuals to retrieve more specific memories. It is possible that retrieval practice of specific memories might weaken associated general memories, contributing to the therapeutic effect of these treatments.

### Limitations and Future Directions

A limitation of the present study is that the specificity of retrieved memories was manipulated by instruction. As such, during the generation of general memories, there were cases in which a specific memory initially came to mind, from which the general memory was abstracted upon a reminder that the memory needed to be general. In such cases, it is possible that participants continued to retrieve the general memory “via” the specific memory for the remainder of the experiment. In addition, the present study only examined RIF in the short term. While previous research has demonstrated enduring RIF effects using neutral words as stimuli (Storm et al., 2012), further research is needed to confirm whether this applies to autobiographical memory.

The current study examined RIF of specific memories in the context of a lab-based experimental paradigm. An important next step will be establishing how such mechanisms operate in the real world. It would be interesting to investigate, for example, whether individual differences in RIF tendency relate to trait-level differences in memory specificity, as has been found with positivity biases in autobiographical memory (Marsh et al., 2019; Storm et al., 2012). Finally, the present study was conducted within a healthy student sample. It will be important to establish whether the same effects can be observed within psychiatric populations.

### Conclusion

The present study found that the repeated retrieval of general memories could induce the forgetting of related specific memories of both positive and negative valence. This forgetting effect generalized to novel retrieval cues, indicating suppression of the memory trace itself. These findings suggest that competition can occur across general and more specific levels of the autobiographical memory hierarchy proposed by Conway and Pleydell-Pearce (2000). Inhibitory control mechanisms recruited to overcome this competition, while facilitating retrieval of the general memory, can impair later recall of the competing specific memories. This inhibition of specific memories may exacerbate OGM tendencies in contexts where there is a preexisting tendency to retrieve general memories in a repetitive manner, such as in depressive rumination. We suggest that the extent to which specific memories are targeted for inhibition during general memory retrieval may depend on the specific retrieval goals. Positive beliefs about rumination and functional avoidance of specific memories may increase the extent to which specific memories are considered an interference, leading to greater and more frequent inhibition of those specific memories. As such, we suggest that tendencies to repetitively retrieve general memories, combined with retrieval goals that favor general memories may trigger inhibition of specific memories. This weakening of specific memories may shape longer-term autobiographical memory availability in favor of more abstract, generalized traces. Further research is needed to confirm that these mechanisms do indeed operate to shape longer-term autobiographical memory in the “real world,” and to what extent this depends on retrieval goals. Nonetheless, these findings extend our understanding of OGM and have potentially important clinical implications.

### Context Paragraph

Although theories explaining OGM in relation to RIF have existed for about 15 years, few studies have experimentally tested the proposed mechanisms. With combined expertise in autobiographical memory and memory suppression, the authors have explored the role of memory suppression in OGM. Several studies have suggested that memory suppression mechanisms may affect the specificity and emotional valence of autobiographical memory, but previously this has been only inferred (Matsumoto & Mochizuki, 2017; Matsumoto et al., 2020) or indirectly correlated (Marsh et al., 2019). The present study was designed to directly examine whether the mechanisms underlying RIF could contribute to OGM tendencies. The findings have theoretical implications in relation to RIF, alongside practical implications for the design of novel psychological therapies targeting autobiographical memory specificity.

## Footnotes

1 The independent rater autobiographical memory categorizations were largely aligned with the experimental instructions to retrieve a general or specific memory, indicating that the proportion of incorrect memory responses was low. However, we conducted a follow-up analysis including only memories which the independent rater categorizations aligned with the experimental instruction. We also excluded cues where participants recalled an incorrect memory, or failed to recall any memory, on at least one of the retrieval practice trials. We used the generalized linear mixed model (glmer function in R; Bates et al., 2015) with a random intercept model, and examined whether the retrieval practice condition (practiced, unpracticed or baseline), cue valence, and their interaction predicted correct specific memory recall on the final recall test. In Experiment 1, the main effect of retrieval practice was significant (*OR* = 0.35, 95% CI [0.20, 0.59], *p* < .001), but the main effects of cue valence (*OR* = 0.88, 95% CI [0.52, 1.48], *p* = .63) and interaction (*OR* = 1.93, 95% CI [0.69, 5.59], *p* = .21) were not significant. Again, in Experiment 2, the main effect of retrieval practice was significant (*OR* = 0.59, 95% CI [0.40, 0.89], *p* = .012), but the main effects of cue valence (*OR* = 0.95, 95% CI [0.63, 1.43], *p* = .82) and interaction (*OR* = 1.07, 95% CI [0.48, 2.40], *p* = .87) were not significant. These results are consistent with the results of our main ANOVA analyses, indicating that the effects were not influenced by the minority of retrieval practice trials on which an incorrect response was generated. For the data in Experiment 3, we used marginal coding to enter three conditions into model. That is, we created a dummy variable to indicate the effect of general memory practice (1 = general memory condition, 0 = color generation condition, −1 = baseline) and a dummy variable to indicate the effect of color generation (0 = general memory condition, 1 = color generation condition, −1 = baseline). The results showed that only the main effect of general memory practice was significant (*OR* = 0.73, 95% CI [0.58, 0.91], *p* = .004). The main effects of cue valence (*OR* = 0.93, 95% CI [0.69, 1.26], *p* = .65) and color generation (*OR* = 0.93, 95% CI [0.76, 1.15], *p* = .51) were not significant, and the interaction between cue valence and general memory (*OR* = 0.69, 95% CI [0.44, 1.06], *p* = .09) and the interaction between cue valence and color generation (*OR* = 1.00, 95% CI [0.66, 1.51], *p* = .99) were not significant.

## Word List in Experiments 1 and 2

**Table:**

Positive words			Negative words		
successful	interesting	happy	failed	bored	sad
entertaining	surprised	relaxed	lonely	hurt	clumsy
relived	cooperative	tender	incompetence	lazy	selfish
lively	kind	truthful	gloomy	tired	rough
polite	gentle	cheerful	pitiable	tense	interfering
free	proud	actively	spiritless	egocentric	impatient
glad	optimistic	honestly	ashamed	discouraged	regret
extroverted	endeavor	reassuring	disappointed	shy	irresolute
satisfied	hopeful	earnest	cowardly	nervous	liar
upbeat	thoughtful	sociable	miserable	passive	inferior
